# Dissolved hydrogen and nitrogen fixation in the oligotrophic North Pacific Subtropical Gyre

**DOI:** 10.1111/emi.412069

**Published:** 2013-06-10

**Authors:** Samuel T Wilson, Daniela A del Valle, Julie C Robidart, Jonathan P Zehr, David M Karl

**Affiliations:** 1 Department of Oceanography, School of Ocean and Earth Science and Technology, University of HawaiiHonolulu, HI, USA; 2 Center for Microbial Oceanography: Research and Education, University of HawaiiHonolulu, HI, USA; 3 Ocean Sciences Department, University of California, Santa CruzCA, USA

## Abstract

The production of hydrogen (H_2_) is an inherent component of biological dinitrogen (N_2_) fixation, and there have been several studies quantifying H_2_ production relative to N_2_ fixation in cultures of diazotrophs. However, conducting the relevant measurements for a field population is more complex as shown by this study of N_2_ fixation, H_2_ consumption and dissolved H_2_ concentrations in the oligotrophic North Pacific Ocean. Measurements of H_2_ oxidation revealed microbial consumption of H_2_ was equivalent to 1–7% of ethylene produced during the acetylene reduction assay and 11–63% of ^15^N_2_ assimilation on a molar scale. Varying abundances of *Crocosphaera* and *Trichodesmium* as revealed by *nifH* gene abundances broadly corresponded with diel changes observed in both N_2_ fixation and H_2_ oxidation. However, no corresponding changes were observed in the dissolved H_2_ concentrations which remained consistently supersaturated (147–560%) relative to atmospheric equilibrium. The results from this field study allow the efficiency of H_2_ cycling by natural populations of diazotrophs to be compared to cultured representatives. The findings indicate that dissolved H_2_ concentrations may depend not only on the community composition of diazotrophs but also upon relevant environmental parameters such as light intensity or the presence of other H_2_-metabolizing microorganisms.

## Introduction

In the surface waters of the tropical and subtropical open ocean, dissolved H_2_ concentrations typically range from 1–3 nmol l^−1^, equivalent to 300–900% supersaturation relative to atmospheric equilibrium (Herr *et al.*, 1984; Conrad and Seiler, 1988; Moore *et al.*, 2009). The magnitude of the dissolved H_2_ pool is determined by the ‘oceanic H_2_ cycle’ which reflects the balance between production and loss processes. As such, the main source of H_2_ is considered to be biological dinitrogen (N_2_) fixation (Herr *et al.*, 1984; Scranton *et al.*, 1987; Moore *et al.*, 2009), whereby N_2_ is reduced to ammonia (NH_3_), as shown in Eq. 1: 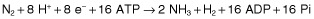
1where ADP and ATP are adenosine-5′-diphosphate and adenosine-5′-triphosphate respectively, H^+^ is hydrogen ion, e^-^ is electron and Pi is inorganic phosphorus (Simpson and Burris, 1984). While N_2_ fixation is more commonly measured than H_2_ production, it is unwise to use the theoretical stoichiometry predicted in Eq. 1 to provide an estimate of H_2_ production associated with nitrogenase activity. This is due to several inherent issues associated with H_2_ cycling linked to N_2_ fixation, as listed below:

(i) Measurements of H_2_ production alongside measurements of N_2_ fixation are always less than the equimolar stoichiometry predicted in Eq. 1 (Schubert and Evans, 1987; Wilson *et al.*, 2010). This is because all diazotrophs contain uptake hydrogenases that re-assimilate a variable portion of H_2_ released during N_2_ fixation to conserve energy (Burns and Hardy, 1975, Tamagnini *et al.*, 2007).
(ii) Rates of net H_2_ production by diazotrophs appear to be highly species-specific. Laboratory-maintained cultures of two diazotrophs, *Crocosphaera* and *Trichodesmium* produce H_2_ at approximately 1% and 25% of their respective rates of N_2_ fixation, as measured by the acetylene reduction (AR) assay (Wilson *et al.*, 2010). The comparatively high rates of net H_2_ production by *Trichodesmium* are a consequence of the cells fixing N_2_ during the daytime as the supply of photosynthetically derived energy and reductant decreases the need to re-assimilate the H_2_ as an energy source, resulting in an increase of net H_2_ production (Wilson *et al.*, 2012b). By comparison, *Crocosphaera* fixes N_2_ during the dark period restricting the supply of cellular energy to nitrogenase from the respiration of photosynthetically fixed carbon (Waterbury *et al.*, 1988; Berman-Frank *et al.*, 2007). This causes a greater demand for the energy and reductant obtained from oxidizing H_2_ and therefore decreases the net H_2_ production (Wilson *et al.*, 2010).
(iii) Field measurements of N_2_ fixation can be conducted using the ^15^N_2_ assimilation technique or the AR assay. The ^15^N_2_ tracer technique is considered to be a measure of net N_2_ fixation as it does not account for dissolved organic and inorganic material released from cells (Montoya *et al.*, 1996; Mulholland *et al.*, 2004). The AR assay measures total nitrogenase activity by quantifying the reduction of acetylene (C_2_H_2_) to ethylene (C_2_H_4_) and therefore represents an indirect assay of N_2_ fixation (Burris, 1975). Because H_2_ production is equimolar with N_2_ fixation (Eq. 1), the AR assay should represent a better measurement when estimating the total amount of H_2_ produced by nitrogenase.

Due to the issues listed above, to define the role of N_2_ fixation in the global H_2_ cycle (e.g. Price *et al.*, 2007), it is imperative to conduct field measurements of both N_2_ fixation and H_2_ production. In this study, simultaneous measurements of N_2_ fixation, biological H_2_ consumption and dissolved H_2_ concentrations were conducted in the surface waters of the open ocean where diazotrophs are present. Results are presented showing the diazotrophic community composition (as measured by *nifH* gene abundance and diversity), rates of net and gross N_2_ fixation (as measured by ^15^N_2_ tracer assimilation and AR assay respectively), H_2_ concentrations and H_2_ oxidation rates (using ^3^H_2_ as a tracer). Quantitative interpretation of the field data is aided by the recent measurement of net H_2_ production and N_2_ fixation in laboratory cultures of diazotrophs to infer the relative contribution of the representative marine N_2_-fixing microorganisms to the oceanic H_2_ cycle.

## Results and discussion

### Sampling overview

The oceanographic cruise was located approximately 250 km north of Oahu, Hawaii in the North Pacific Subtropical Gyre (NPSG) and occurred between 6 and 21 September 2011. The sampling stations were occupied along the north-western edge of an anti-cyclonic eddy spanning a total distance of 90 km and the subsequent westward section of the cruise track which spanned 80 km. Vertical profiles of dissolved H_2_ were conducted daily alongside biogeochemical and hydrographic measurements. Biological rate measurements of N_2_ fixation and H_2_ consumption were conducted at three sampling stations: Station (Stn) 3, 7 and 13 which were sampled on 7, 9 and 18 September 2011 respectively. Descriptions of the hydrographic conditions and biogeochemical properties of the water column are available in the accompanying Supporting Information and also online at http://hahana.soest.hawaii.edu/cmorebiolincs/biolincs.html.

### Dissolved H_2_ concentrations

**Fig 1:**
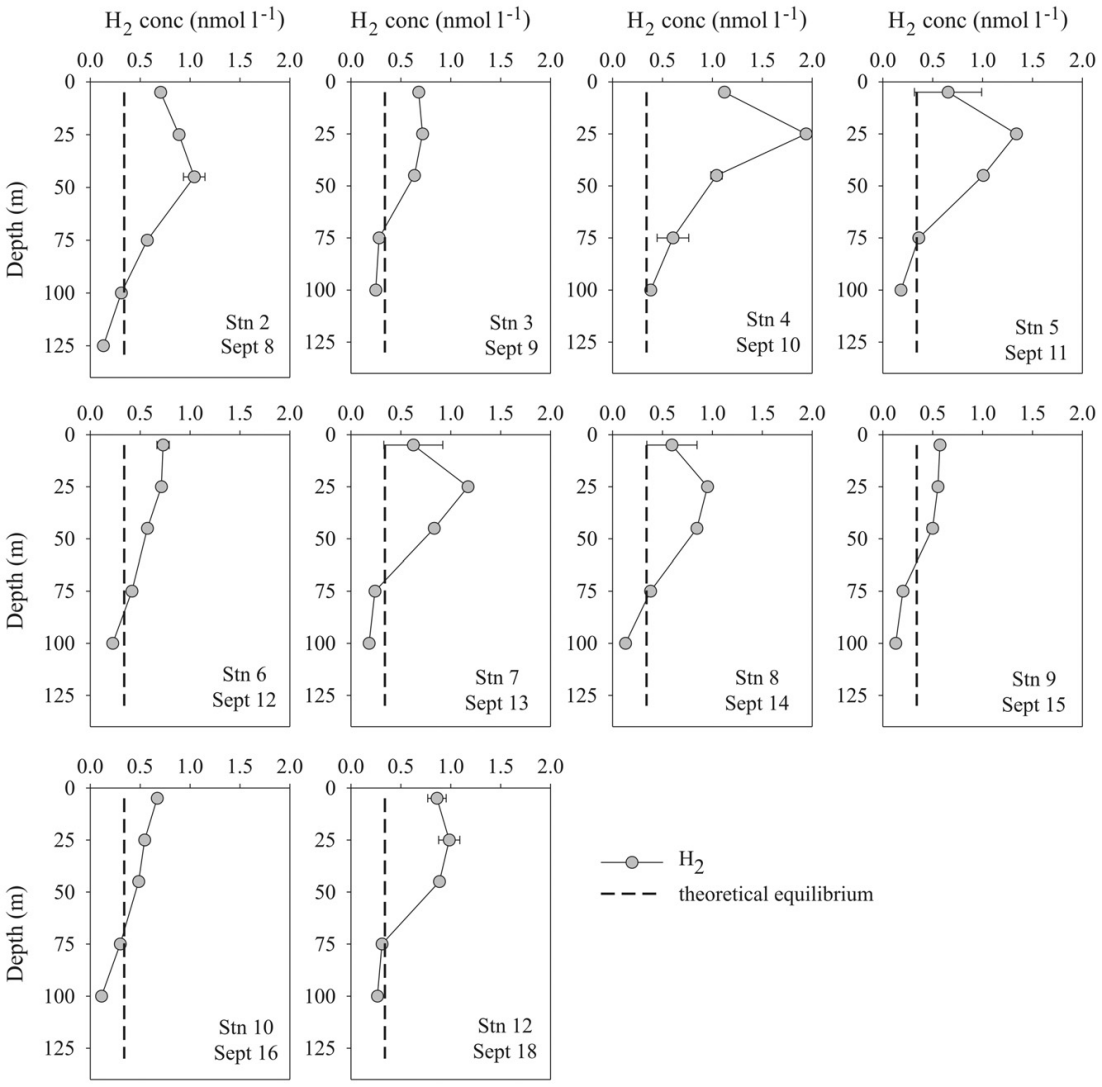
Dissolved H_2_ concentrations (nmol l^−1^) between depths of 5–125 m in the North Pacific Ocean. For each sampling occasion, seawater samples were collected at 1300 h. The theoretical value of dissolved H_2_ concentrations in seawater at atmospheric equilibrium (with an atmospheric concentration of 0.5 ppmv) is represented by the dashed line. Error bars where shown represent standard deviation (*n* = 3).

Dissolved H_2_ concentrations were supersaturated with respect to atmospheric equilibrium in the upper 75 m of the water column (Fig. 1). Overall, dissolved H_2_ concentrations in the surface mixed layer (0–45 m) ranged from 0.5–1.9 nmol l^−1^, with an average concentration of 0.83 nmol l^−1^, equivalent to 250% supersaturation. Dissolved H_2_ concentrations in seawater were calculated using the Bunsen solubility coefficients provided by Wiesenburg and Guinasso (1979). On four separate occasions, the concentrations of dissolved H_2_ in the mixed layer exceeded 1 nmol l^−1^ (Fig. 1). The concentrations of H_2_ measured in surface seawater during this cruise are consistent with measurements in other marine environments (e.g. the Mediterranean Sea, Atlantic and Pacific Ocean) revealing a persistent supersaturation of dissolved H_2_ in the near-surface seawater (Scranton *et al.*, 1982; Herr *et al.*, 1984; Conrad and Seiler, 1988; Moore *et al.*, 2009). At depths exceeding 75 m, a progressive depletion in H_2_ concentrations was observed with values approaching undersaturation with respect to atmospheric equilibrium by a depth of 100 m. Vertical profiles of N_2_ fixation in the NPSG measured on previous occasions (Grabowski *et al.*, 2008; Church *et al.*, 2009) similarly show a decrease at 75 m, consistent with the hypothesis that the dissolved H_2_ is derived from nitrogenase activity.

### N_2_ fixation

**Fig 2:**
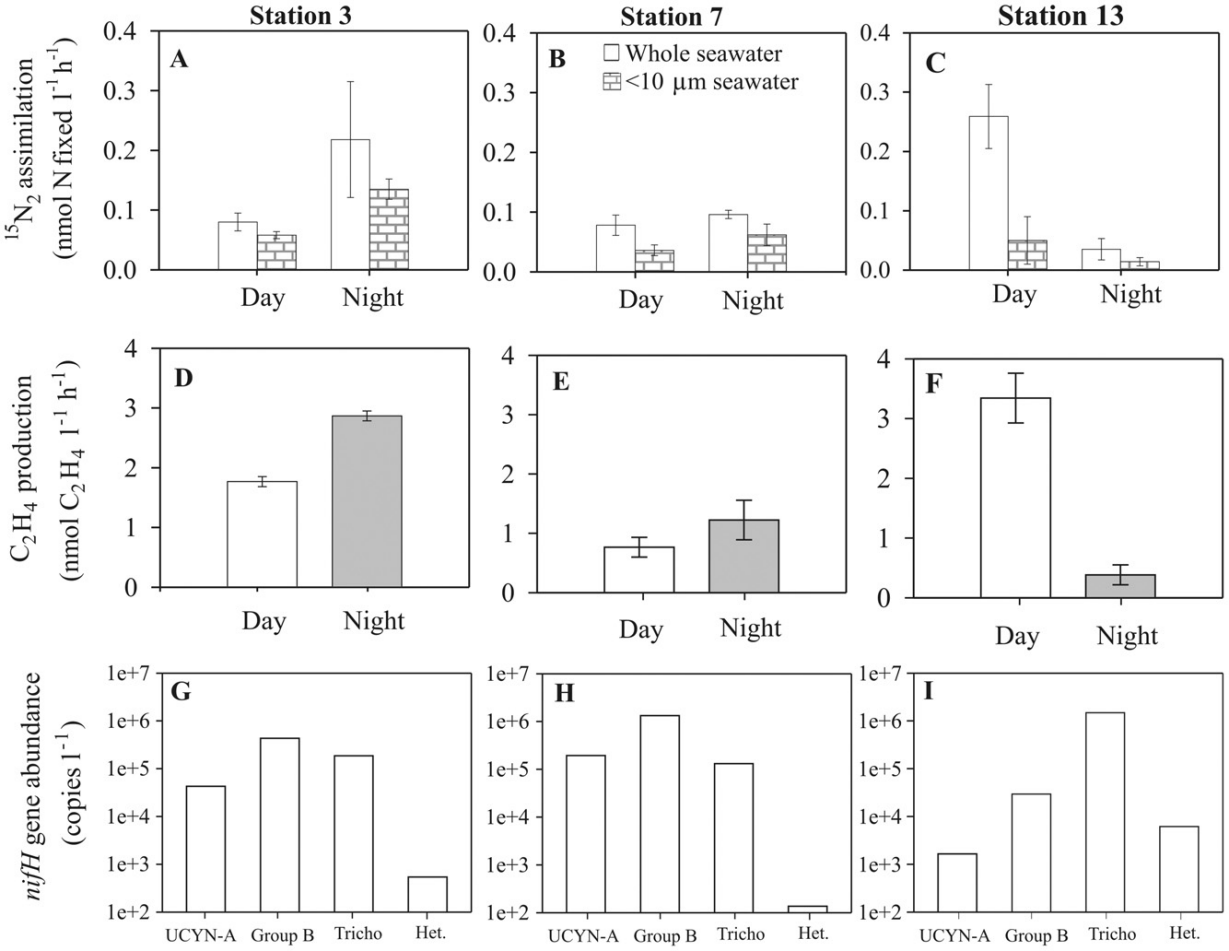
N_2_ fixation rates as measured by (A–C) ^15^N_2_ tracer assimilation and (D–F) the AR assay for seawater samples collected at 25 m and incubated onboard the ship during either the day or night period. Post-incubation size fractionation was conducted for replicate ^15^N_2_ tracer additions and not for the AR assay. The error bars in A–F represent standard error (*n* = 3). The *nifH* gene abundances collected from the same depth on the same date are shown for UCYN-A, Group B (*Crocosphaera* spp.), (Tricho) *Trichodesmium* and (Het) heterocystous cyanobacteria (G–I).

N_2_ fixation rate measurements, determined by both the ^15^N_2_ tracer assimilation and the AR assay, were conducted at Stn 3, 7 and 13. The overall temporal pattern of N_2_ fixation changed between the stations from an initial prevalence during the night-time, to a subsequent dominance during the day-time. Specifically, rates of ^15^N_2_ assimilation during the night-time (0.22 nmol l^−1^ h^−1^) exceeded the day-time (0.08 nmol l^−1^ h^−1^) at Stn 3 (Fig. 2A). In contrast, at Stn 13, rates of ^15^N_2_ assimilation in whole seawater were highest (0.26 nmol l^−1^ h^−1^) during the day-time, compared to the rates during the night-time (0.04 nmol l^−1^ h^−1^) (Fig. 2C). No significant difference was observed between the daytime and night-time measurements of N_2_ fixation at Stn 7. At all sampling stations, the rate of ^15^N_2_ assimilation in whole seawater samples exceeded the comparative rates in the accompanying < 10 μm size-fractionated seawater samples. Comparison of the < 10 μm size-fraction across the three stations reveals low variability in the rate of ^15^N_2_ assimilation (0.04–0.06 nmol l^−1^ h^−1^) during the daytime. In contrast, night-time rates of ^15^N_2_ assimilation for the < 10 μm size fraction varied by an order of magnitude, decreasing from 0.14 nmol l^−1^ h^−1^ at Stn 3, to 0.01 nmol l^−1^ h^−1^ at Stn 13 (Fig. 2A–C).

AR was measured on whole seawater samples, and a significant increase in C_2_H_4_ concentrations was always detected during the 3–4 h incubations (Fig. 2D–F). The rates of C_2_H_4_ production support the ^15^N_2_ assimilation measurements with higher rates during the night-time (2.9 nmol l^−1^ h^−1^) compared to the daytime (1.8 nmol l^−1^ h^−1^) at Stn 3. Furthermore, at Stn 13, the diel pattern of C_2_H_4_ production changed with daytime (3.3 nmol l^−1^ h^−1^) exceeding night-time (0.4 nmol l^−1^ h^−1^) (Fig. 2F). Overall, the ratio of C_2_H_4_ to ^15^N_2_ assimilation varied from 9–22 which exceeds the theoretical ratio of 3:1 (Capone, 1993) by 3- to 7-fold. It should be noted that the theoretical ratio of 3:1 is based on the difference between two electrons required to reduce C_2_H_2_ to C_2_H_4_, and six electrons needed to reduce N_2_ to 2NH_3_. The reasons for the discrepancies between the theoretical and observed ratios have previously been discussed (e.g. Graham *et al.*, 1980) and focus mainly on the excretion of N from the cell and the role of H_2_. There is insufficient data in this study to contribute to this discussion; however we do note from our work and the relevant literature that there is a greater difference in the AR:^15^N_2_ assimilation ratio in field measurements compared to culture-based analyses. Furthermore, there is a lack of experimental testing on the effect of key environmental parameters on the AR:^15^N_2_ assimilation ratio, e.g. light intensity or nutrient concentrations (Mague *et al.*, 1977).

### Diazotroph community structure

Representative N_2_ fixing microorganisms in the open ocean include: (i) the filamentous, non-heterocystous cyanobacterium *Trichodesmium*, (ii) the heterocystous cyanobacteria (e.g. *Richelia* and *Calothrix*) that form symbioses with eukaryotic algae, and (iii) unicellular cyanobacteria including Group A (termed UCYN-A) and Group B (e.g. *Crocosphaera*) (Mague *et al.*, 1977; Carpenter and Romans, 1991; Zehr *et al.*, 2001). The analysis of *nifH* gene abundances revealed Group B was the most abundant diazotroph for the first two sampling occasions (Stns 3 and 7), with 4.3 × 10^5^ and 1.3 × 10^6^ gene copies l^−1^. At the third sampling site (Stn 13), *nifH* gene copies of Group B decreased to 2.9 × 10^4^ gene copies l^−1^, in contrast to *Trichodesmium nifH* gene copies which increased to a maximum of 1.6 × 10^6^ gene copies l^−1^ (Fig. 2). The shift from a Group B-dominated to a *Trichodesmium*-dominated diazotroph community between Stn 3 and Stn 13 respectively, could account for the change in the diel pattern of N_2_ fixation. The unicellular *Crocosphaera* fixes N_2_ during the night, and rates of N_2_ fixation were highest during the night-time in waters where *Crocosphaera* gene copies were most abundant. Two other groups of diazotrophs were present at lower abundances throughout the cruise; UCYN-A *nifH* abundance ranged from 1.6 × 10^3^ to 1.9 × 10^5^ gene copies l^−1^, and the total heterocystous cyanobacterial gene copies were the lowest of all *nifH* gene groups measured with a maximum abundance of 6.2 × 10^3^ gene copies l^−1^ at Stn 13.

### Microbial consumption of H_2_

Biological ^3^H_2_ oxidation was measured during the day-time and night-time, alongside N_2_ fixation rate measurements at Stns 3 and 13. Overall, the rates of biological ^3^H_2_ oxidation ranged from 15 to 42 pmol H_2_ l^−1^ h^−1^ (Table 1). At Stn 3, night-time rates of biological ^3^H_2_ oxidation (25 pmol H_2_ l^−1^ h^−1^) exceeded daytime rates (15 pmol H_2_ l^−1^ h^−1^) by 66%. In contrast, at Stn 13 the daytime rates of biological ^3^H_2_ oxidation (42 pmol H_2_ l^−1^ h^−1^) were 68% higher than night-time (25 pmol H_2_ l^−1^ h^−1^) (Table 1). In this respect, the diel variability in biological ^3^H_2_ oxidation rates reflect the diel patterns observed in the rate of ^15^N_2_ assimilation and the AR assay. The measured rates of ^3^H_2_ oxidation were equivalent to 11–63% of ^15^N_2_ assimilation and 1–7% of C_2_H_4_ production as measured by the AR assay.

**Table 1:** Rates of biological ^3^H_2_ oxidation conducted on whole seawater samples collected at 25 m (the error bars represent standard deviation of replicate samples, *n* = 3). The rate measurements are compared with the ^15^N_2_ assimilation and C_2_H_4_ production values in whole seawater (Fig. 1) to calculate the percentage of N_2_ fixation accounted for by biological oxidation

Station sampled	Water-column ^3^H_2_ oxidation (pmol H_2_ L^−1^ h^−1^)	% of AR assay accounted for by ^3^H_2_ oxidation	% of ^15^N_2_ assimilation accounted for by ^3^H_2_ oxidation	Turnover time of dissolved H_2_ pool (h)
Stn 3 (Day)	15 ± 1	0.8	18.8	40
Stn 3 (Night)	25 ± 4	0.9	11.4	23
Stn 13 (Day)	42 ± 6	1.3	16.2	22
Stn 13 (Night)	25 ± 2	6.6	62.5	36

Previous measurements of biological H_2_ consumption have been reported from other aquatic habitats including coastal seawater (Punshon *et al.*, 2007), shallow lakes (Conrad *et al.*, 1983) and river systems (Paerl, 1982). These previous studies have revealed H_2_ turnover times ranging from < 1 h in a eutrophic shallow lake (Conrad *et al.*, 1983) to 2–3 days in high-latitude coastal seawater (Punshon *et al.*, 2007). In comparison, the H_2_ turnover times measured in this study at two sampling stations ranged from 22–40 h (Table 1).

### Estimation of the production and consumption of H_2_ associated with N_2_ fixation

The measured rates of N_2_ fixation using the AR assay at Stns 3 and 13 were used to estimate the production of H_2_ derived from nitrogenase (Table 2). We use laboratory-derived measurements of net H_2_ production by *Trichodesmium* and *Crocosphaera* cultures described in the Introduction to provide upper and lower boundaries for net H_2_ production. Therefore in contrast to Price and colleagues (2007) who estimated net H_2_ production at 55% of N_2_ fixation in the marine environment, we set maximum and minimum net H_2_ production rates at 25% and 1% of C_2_H_4_ production respectively. The resulting estimates of net H_2_ production range from 0.004 to 0.84 nmol H_2_ l^−1^ h^−1^ in the upper water column. Furthermore, the calculations indicate that N_2_ fixation can replenish the dissolved H_2_ pool in as little as 1 h and extending up to 34 h, with the exception of 19 September during the night-time which has an excessively long upper estimate of 245 h (Table 2).

**Table 2:** Estimation of H_2_ production in the open ocean water column at a depth of 25 m. The minimum and maximum values are based on 1% and 25% of C_2_H_4_ production

Date sampled	Water-column H_2_ concentration (nmol H_2_ L^−1^)	AR assay (nmol C_2_H_4_ L^−1^ h^−1^)	Estimated H_2_ prod. (nmol H_2_ L^−1^ h^−1^)		Estimated time to replenish H_2_ stock (h)
			Min.	Max.	
Stn 3 (day)	0.6	1.77	0.018	0.44	1–34
Stn 3 (night)	0.6	2.87	0.029	0.72	1–21
Stn 13 (day)	0.93	3.34	0.033	0.84	1–28
Stn 13 (night)	0.93	0.38	0.004	0.10	10–245

The estimates of net H_2_ production in surface seawater as listed in Table 2 can be compared with the biological ^3^H_2_ oxidation measurements which were conducted on the same seawater samples (Table 1). The rates of ^3^H_2_ oxidation were equivalent to 0.8–6.6% of the AR assay (Table 1) indicating biological consumption was equivalent to the lower end of estimated rates of net H_2_ production (i.e. comparable to rates of net H_2_ production by *Crocosphaera*). This suggests that concentrations of dissolved H_2_ may increase in the presence of *Trichodesmium* and stimulate the diel cycle of H_2_ in surface seawater as observed by Herr and colleagues (1984) in the South Atlantic. However in this study, the increase in *Trichodesmium* abundance was not matched by an increase in net H_2_ concentrations (Fig. 1) suggesting that field populations of *Trichodesmium* may re-assimilate more of the H_2_ produced via nitrogenase compared to their cultured counterparts and are therefore more energetically efficient. Alternatively, other sinks of H_2_ in the upper ocean may contribute to the loss of dissolved H_2_, and these are considered in the next section.

### H_2_ cycling in the open ocean

The oceanic H_2_ cycle depends not only on biological production and consumption as discussed with reference to diazotrophs, but also physical forcing mechanisms. The physical processes can be considered with respect to the sink terms for H_2_, comparing estimates of air–sea gas exchange and downwards diffusion with biological oxidation. The downward diffusion of H_2_ can be estimated from the concentration gradient between depths of 45 m and 75 m, using the vertical eddy diffusion coefficient reported by Ledwell *et al*. (1993) (Table 3). The flux of H_2_ to the atmosphere can be estimated according to Eq. 2, where *S* is the Bunsen solubility coefficient (Wiesenburg and Guinasso, 1979), Δ*p* is the difference in partial pressure (*p*) between the atmosphere and ocean, and *k* is the transfer velocity. An atmospheric H_2_ concentration of 0.53 parts per million by volume (ppmv) was used in the flux calculations (Novelli *et al.*, 1999). The transfer velocity (*k*) was calculated according to Wanninkhof (1992) (Eq. 3) where *U* is the wind speed (m sec^−1^) normalized to 10 m above the sea surface and *Sc* represents the Schmidt number for H_2_ at in situ seawater temperature and salinity (Jähne *et al*., 1987). 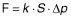
2

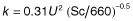
3

**Table 3:** Depth integrated (0–45 m) inventories of dissolved H2 concentrations in comparison with sea-air gas flux, downward diffusion, and estimated biological consumption.

Date	Depth-integrated (0–45 m) H_2_ inventories (μmol m^−2^)	Water column sea–air H_2_ flux (μmol H_2_ m^−2^ h^−1^)	Downward diffusion (μmol H_2_ m^−2^ h^−1^)	Biological consumption (μmol H_2_ m^−2^ h^−1^)
Stn 3 (day)	30.6	0.03–0.06	0.42	0.03–5.17
Stn 3 (night)	30.6	0.04–0.08	0.42	0.31–3.87
Stn 13 (day)	41.0	0.11–0.37	0.68	0.47–5.80
Stn 13 (night)	41.0	0.08–0.33	0.68	1.69–16.05

To obtain depth-integrated estimates of H_2_ consumption, we used recent measurements of N_2_ fixation profiles at Stn ALOHA (HOT cruises #202–213, corresponding to June 2008–July 2009) to calculate the relationship between N_2_ fixation measurements at 25 m and 0–45 m depth integrated values (y = 46.12x + 23.8, r^2^ = 0.82). The conversion factor was applied to the rates of N_2_ fixation (Fig. 1) using the percentage of AR assay and ^15^N_2_ assimilation (Table 1) to provide a lower and upper estimate of biological H_2_ consumption respectively, integrated across the 0–45 m depth horizon. While there is approximately an order of magnitude difference between the upper and lower estimates of biological consumption (Table 3), the median values for turnover times compare favourably with the rates of H_2_ consumption calculated from the ^3^H_2_ oxidation measurements for discrete seawater samples collected from 25 m (Table 1). It is evident that for this time period, biological consumption and downward diffusion represented the main loss pathways for dissolved H_2_ in the upper ocean. The estimated flux of H_2_ to the atmosphere ranged from 0.03–0.33 μmol m^−2^ h^−1^ (Table 3) and should be considered a low estimate of H_2_ loss to the overlying atmosphere due to the predominantly low wind speeds (< 5 m sec^−1^) during the cruise.

## Conclusion

During a 10-day sampling period in the NPSG, dissolved H_2_ concentrations were 147–560% supersaturated with respect to atmospheric equilibrium. Measured rates of ^15^N_2_ assimilation and AR revealed a change in the prevalence of N_2_ fixation from night-time to day-time, which was accompanied by a decrease in the abundance in Group B *nifH* gene copies, and an increase in the abundance of *Trichodesmium nifH* gene copies. Prior to this study, it was hypothesized that varying abundance of larger, daytime N_2_ fixing microorganisms (e.g. *Trichodesmium*) might influence the dissolved pool of H_2_ in surface seawater due to their relatively high rates of net H_2_ production (Wilson *et al.*, 2010). However, the absence of varying dissolved H_2_ concentrations indicate that field populations of *Trichodesmium* may be more efficient at recycling H_2_ compared to laboratory cultures. Biological H_2_ oxidation measurements in seawater sampled from 25 m depth indicate that H_2_ production needed to exceed 1–6% of C_2_H_4_ production to cause an increase in the ambient pool of dissolved H_2_ (Table 1). This is considerably lower than in laboratory-maintained *Trichodesmium* cultures where the rate of net H_2_ production was equivalent to 25% of C_2_H_4_ production (Wilson *et al.*, 2012b). Using either the AR assay or the ^15^N_2_ assimilation technique caused approximately one order of magnitude variability when calculating the efficiency of H_2_ cycling. We consider the AR assay to be more representative of nitrogenase activity but recognize that it is an indirect measurement and not widely used in oceanographic studies on non-concentrated seawater samples. Comparison of the loss mechanisms for dissolved H_2_ in the upper ocean indicated that biological oxidation represented the most prevalent sink compared to downward diffusion and flux to the atmosphere (Table 3).

It should be noted that oceanic H_2_ cycling is not limited to diazotrophs, and opportunistic H_2_-oxidizing microorganisms (e.g. aerobic anoxygenic photosynthetic bacteria and heterotrophic bacteria) will also metabolize H_2_. Furthermore, other sources of H_2_ such as photochemical degradation of dissolved organic matter (Punshon and Moore, 2008) and fermentation (Schropp *et al.*, 1987) should be considered when studying H_2_ cycling in the upper water column. Nonetheless, this study provides an important contribution to our understanding on the role of diazotrophs in dissolved H_2_ cycling and reveals it to be more restrained than measurements conducted using laboratory cultures of diazotrophs.

## Experimental procedures

Dissolved H_2_ concentrations were measured with a reduced gas analyzer (Peak Laboratories, Mountain View) adapting the method of Moore and colleagues (2009). The rate of H_2_ consumption was quantified by measuring the production of ^3^H_2_O from tracer additions of ^3^H_2_ as previously used in laboratory cultures of diazotrophs (Chan *et al.*, 1980) and environmental microbial assemblages (Paerl, 1983). To determine the rate of N_2_ fixation, measurements of ^15^N_2_ assimilation and AR were carried out as described in Wilson and colleagues (2012a). The *nifH* gene abundance was quantified using the methodological protocols previously published by Moisander and colleagues (2010). Full descriptions of all the analytical methods for measuring H_2_ and N_2_ fixation and also the accompanying hydrographic datasets are in the Supporting Information (see Appendix S1).

## Supporting Information

**Appendix S1.** The relevant hydrographic and biogeochemical datasets together with full descriptions of the analytical methods for measuring dissolved H_2_ and N_2_ fixation are in the Supporting Information.

**Figure S1.** 14-day composite of satellite derived SSHA 100 km north of the Hawaiian Islands in the Pacific Ocean between 7 and 21 September 2011 (data from Moderate Resolution Imaging Spectroradiometer). A summary of the cruise transect is indicated by the solid black line and the labeled white circles represent the sampling stations discussed in the text. Station ALOHA, the long-term sampling station for the Hawaii Ocean Time-series (HOT) programme, located at 22°45′N, 158°W is also highlighted.

**Figure S2.** Representative water column profiles for the two sections of the cruise track, (A-B) Stn 3 and (C-D) Stn 13.
