# Supplementary material for: Dissolved hydrogen and nitrogen fixation in the oligotrophic North Pacific Subtropical Gyre
Source: Environ Microbiol Rep. 2013 Jun 10;5(5):697–704. doi: 10.1111/emi.412069 (PMC4271820; doi:10.1111/emi.412069)
Supplement: Supplementary file 1 — Appendix S1. The relevant hydrographic and biogeochemical datasets together with full descriptions of the analytical methods for measuring dissolved H2 and N2 fixation are in the Supporting Information. [file emi40005-0697-SD1.doc]

**Supplementary Information**

*Water column structure and biogeochemical properties*

Shipboard sampling was conducted in the North Pacific Subtropical Gyre along a cruise track which transited the edge of two anticyclonic mesoscale eddies (Fig S1). A total of 11 sampling stations were occupied during the cruise, spanning a total distance of approximately 170 km. To characterize the upper water column, vertical profiles were conducted using a Conductivity-Temperature-Depth (CTD) system coupled to a rosette consisting of 24 x 12 liter Niskin-like ‘Bullister’ bottles. Oxygen (O_2_) and fluorescence sensors were calibrated against discrete measurements of dissolved O_2_ (Carritt and Carpenter, 1966) and chlorophyll *a* (chl *a*) extracted and analyzed by fluorometry (Turner AU-10). Seawater for determination of nutrient concentrations (NO_2_^-^ + NO_3_^-^, SRP, and Si) was subsampled into acid washed 125 ml polyethylene bottles, capped, and then stored frozen. Sample analysis was performed on land as documented in the online manual for “HOT Laboratory Protocols” ([*http://hahana.soest.hawaii.edu*](http://hahana.soest.hawaii.edu/)).

An overview of the water-column biogeochemistry is provided by comparing vertical profiles of nutrients and chl *a* from Stn 3 (24^o^ 43.4′ N, 157^o^ 33.2′ W) during the first part of the transect and from Stn 13 (24^o^ 48′ N, 158^o^ 15.2′ W) during the latter part of the transect (Fig. S1). At Stn 3, the maximum chl *a* concentrations were observed at a depth of 115 m, compared to a depth of 105 m at Stn 13 (Fig. 2). The nutrient profiles revealed a significant difference between concentrations of silicate (Si) (one-tailed t-test, P=0.04) and soluble reactive phosphorus (SRP) (one-tailed t-test, P=0.03) in the surface mixed layer (0–45m) between Stn 3 and 13. Furthermore, the vertical profile of SRP concentrations at Stn 3 revealed a distinct subsurface minimum with concentrations decreasing from 0.09 µM at 25 m to 0.02 µM at 100 m. Beneath 100 m, the concentrations of nitrate + nitrite (NO_3_^-^ + NO_2_^-^), Si, and SRP increased more rapidly with depth at Stn 13 where concentrations were 16, 42, and 210% higher than Stn 3 by 175 m, respectively (Fig. S2).

*H_2_ measurements*

Discrete seawater samples for measuring dissolved H_2_ concentrations were collected into acid-washed, glass-stoppered 300 ml Wheaton bottles. Samples were analyzed immediately after collection with a total sample processing time of <2 h. To quantify H_2_ concentrations, seawater was sub-sampled from the Wheaton bottles into a 50 ml glass syringe (Perfektum) via 1/8˝ polyetheretherketone (PEEK) tubing. The syringe was flushed twice with sample water, ensuring the last flush was free of air bubbles. A custom-built syringe actuator ensured that a consistent volume of seawater (35 ml) was always introduced into the syringe. Subsequently 5 ml of H_2_-free (<10 parts per trillion) air (Airgas) was introduced into the syringe, H_2_ was extracted from the seawater using headspace equilibration, and the headspace was subsequently injected into the gas analyzer (described below). To prevent accidental addition of seawater following after the headspace injection, the sampling inlet for the analyzer was fitted with a hydrophobic syringe filter (13 mm PTFE membrane, 0.2 µm pore size).

We note that the samples are not preserved and therefore H_2_ concentrations could potentially change between the time of collection and analysis. The likelihood of this occurring in oligotrophic seawater samples within < 2 h of sample collection is considered minimal. The analysis of replicate samples in random order on previous occasions did not result in any significant increase in the standard deviation of replicate (typically 3) seawater samples. Also whilst this study shows that production of H_2_ via N_2_ fixation can replenish the dissolved H_2_ pool in 1-38 h, we consider the upper estimate of 1 h to be high and the median value of 18 h to be more reasonable which exceeds the 1-2 h required for processing all samples.

H_2_ was quantified with a reduced gas analyzer that couples a mercuric oxide (HgO) bed to a reducing compound photometer (Peak Laboratories, USA). The stoichiometric reduction of HgO by H_2_ gas releases mercury vapor which is quantified using an ultraviolet absorption photometer located immediately downstream of the HgO bed. For safety purposes, the gas flow exiting the detector passes through an activated charcoal mercury vapor scrubber before venting to the atmosphere. Prior to the detector, the carrier gas (Ultra High Purity air) passes through two analytical columns maintained at 104 ^o^C. The first column is packed with Unibeads 1S (60/80 mesh, 0.32 cm diameter and 41.9 cm length), and the second column with Molecular Sieve 13X (60/80 mesh, 0.32 cm diameter and 206 cm length). The analytical precision based on the comparison of 4 samples at atmospheric equilibrium (0.3 nmol l^-1^) was ± 2%. The analyzer was calibrated using a 1 ppmv H_2_ standard (Scott Marrin) that was diluted up to 100-fold using zero-H_2_ air. The concentration of dissolved H_2_ in seawater was calculated according to the Bunsen solubility coefficients provided by Wiesenburg and Guinasso (1979).

On two separate occasions, the rate of H_2_ consumption was quantified by measuring the production of ^3^H_2_O from tracer additions (0.024–0.046 nM) of ^3^H_2_. This method has previously been used to measure ^3^H_2_ uptake in laboratory cultures of diazotrophs (Chan *et al*., 1980) and environmental microbial assemblages (Paerl, 1983). The seawater sample was collected in a 40 ml borosilicate glass vial with 2–3 times overflow and sealed with no headspace using Teflon-faced butyl rubber stoppers. Tritium gas (specific activity: 2 TBq/mmol; ViTrax, California) was injected into the vial in tracer quantities (10 to 25 pM) and shaken before quickly venting non-dissolved ^3^H_2_ in a fume hood. Seawater samples amended with ^3^H_2_ were incubated for 4 h in the deckboard incubators at repeated intervals during the day and night periods of a diel cycle. Samples were analyzed in triplicate with control samples for abiotic conversion of ^3^H_2_ consisting of 0.2 µm filtered seawater. No activity was observed in the control samples during the experiments. At the end of the incubation, a 1 ml sub-sample was removed using a syringe and injected into a scintillation vial containing scintillation cocktail (Ultima Gold LLT, Perkin Elmer) and counted immediately in a liquid scintillation analyzer (Tri-Carb 2910 TR, Perkin Elmer) to determine the total activity added to the sample. To quantify the amount of transformed ^3^H_2_, a separate 2 ml subsample was added to a scintillation vial and purged with N_2_ (100 ml min^-1^ for 3 min) in a fume hood to remove any remaining ^3^H_2_. A 1 ml aliquot of the sparged samples was subsequently pipetted into a second scintillation vial containing liquid scintillation cocktail and counted. To account for isotopic discrimination effects when calculating the rate of H_2_ oxidation, we used the fractionation factor reported in Soffiento *et al*. (2006). It should be noted that as acknowledged by Soffiento *et al*. (2006), fractionation effects may vary between the different hydrogenase enzymes *e.g*. iron(Fe)-only hydrogenase compared to the nickel-iron (NiFe) hydrogenases contained by cyanobacteria (Tamagnini *et al*., 2007). This should be resolved by analyzing the fractionation factor in phylogenetically distinct hydrogenase-containing microorganisms before assessing the consequences of measuring ^3^H_2_ oxidation in mixed microbial assemblages.

*N_2_ fixation rate measurements*

Rates of N_2_ fixation were measured using both the ^15^N_2_ tracer technique and the acetylene reduction (AR) assay at three sampling stations: Stn 3, 7, and 13, which were occupied on the 9, 13, and 19 September, respectively. The AR assay was conducted using a reduced gas analyzer, similar to the instrument described in ‘*H_2_ measurements*’, for the quantification of C_2_H_4_ production (Wilson *et al*., 2012). The increased sensitivity (5 pmol l^-1^) provided by the reducing compound photometer compared to standard C_2_H_4_ quantification using gas chromatography-flame ionization detector (GC-FID) permits the AR assay to be conducted on seawater samples with no preconcentration of the biomass. Control treatments consisted of 0.2 µm filtered surface seawater, analyzed in triplicate alongside the regular seawater samples. The blank to signal ratio, indicative of the biological production relative to the background presence of C_2_H_4_ ranged from 75–82%. Both samples and controls were incubated using deckboard incubators with typical incubation periods of 3–4 h.

Alongside the AR assay the rate of ^15^N_2_ assimilation into particulate biomass was also measured in seawater samples. The ^15^N_2_ tracer was added to seawater samples as ‘^15^N_2_ enriched seawater’, prepared onboard the ship by filtering seawater collected from 25 m through a 0.2 µm filter, followed by vacuum degasification (250 mbar for 40 min). The ^15^N_2_ gas (98% purity; Isotech Laboratories, Inc.) was dissolved in the sterile, degassed seawater and 50 ml of ^15^N_2_ enriched seawater was added to the seawater samples in 4.3 liter polycarbonate bottles to give a final ^15^N_2_ enrichment of 1.5 atom%. Samples were incubated in the presence of ^15^N_2_ tracer for either 11 h or 13 h corresponding to the day/night-time, respectively. Seawater samples designated for night-time analysis were collected at the same time as the day-time samples and incubated without tracer additions until spiked with ^15^N_2_ enriched seawater at 2000 hrs. Post-incubation, the seawater samples were filtered onto combusted 25 mm glass fiber filters as both unfiltered (whole) seawater and the <10 µm size fraction (representing UCYN-A and Group B). The samples were then stored at -20^o^C prior to analysis on land to quantify the ^15^N_2_ enrichment of particulate material using an elemental analyzer-isotope ratio mass spectrometer, as described in Montoya *et al*. (1996).

*Molecular analysis of nifH*

Discrete seawater samples (2–4 liters) were collected at 1300 hrs using the CTD-rosette from a depth of 25 m, filtered using a peristaltic pump through a 0.22 µm Sterivex filter (Millipore, Billerica, MA, USA) and stored in liquid N_2_. A full description of methodological protocols including DNA extraction and quantitative PCR analyses has been previously published by Moisander *et al*. (2010).

References

Carritt, D.E., and Carpenter, J.H. (1966) Comparison and evaluation of currently employed modifications of the Winkler method for determining dissolved oxygen is seawater: a NASCO report. *J Mar Res* **24**: 286–318.

Chan, Y.K., Nelson, L.M., and Knowles, R. (1980) Hydrogen metabolism of *Axospirillum brasilense* in nitrogen-free medium. *Can J Microbiol* **26**: 1126–1131.

Moisander, P.H., Beinart, R.A., Hewson, I., White, A.E., Johnson, K.S., Carlson, C.A., Montoya, J.P., and Zehr, J.P. (2010) Unicellular cyanobacterial distributions

broaden the oceanic N_2_ fixation domain. *Science* **327**:1512–14.

Montoya, J.P., Voss, M., Kähler, P., and Capone, D.G. (1996) A simple, high-precision,

high-sensitivity tracer assay for N_2_ fixation. *Appl Environ Microbiol* **62**: 986–993.

Paerl, H.W. (1983) Environmental regulation of H_2_ utilization (^3^H_2_ exchange) among natural and laboratory populations of N_2_ and non-N_2_ fixing phytoplankton. *Microb Ecol* **9**: 79–97.

Soffientino, B., Spivack, A.J., Smith, D.C., Roggenstein, E.B., and D’Hondt, S. (2006) A versatile and sensitive tritium-based radioassay for measuring hydrogenase activity in aquatic sediments. *J Microbiol Methods* **66**: 136–146*.*

Wiesenburg, D.A., and Guinasso, N.L. (1979) Equilibrium solubilities of methane, carbon monoxide and hydrogen in water and seawater. *J Chem Eng Data* **24**: 356–360.

Wilson, S. T., Böttjer, D., Church, M.J., and Karl, D.M. (2012) Comparative assessment of nitrogen fixation methodologies conducted in the oligotrophic North Pacific Ocean. *Appl Environ Microbiol* **78**: 6516–6523.
