# Supplementary figures and images for: Dissolved hydrogen and nitrogen fixation in the oligotrophic North Pacific Subtropical Gyre
Source: Environ Microbiol Rep. 2013 Jun 10;5(5):697–704. doi: 10.1111/emi.412069 (PMC4271820; doi:10.1111/emi.412069)

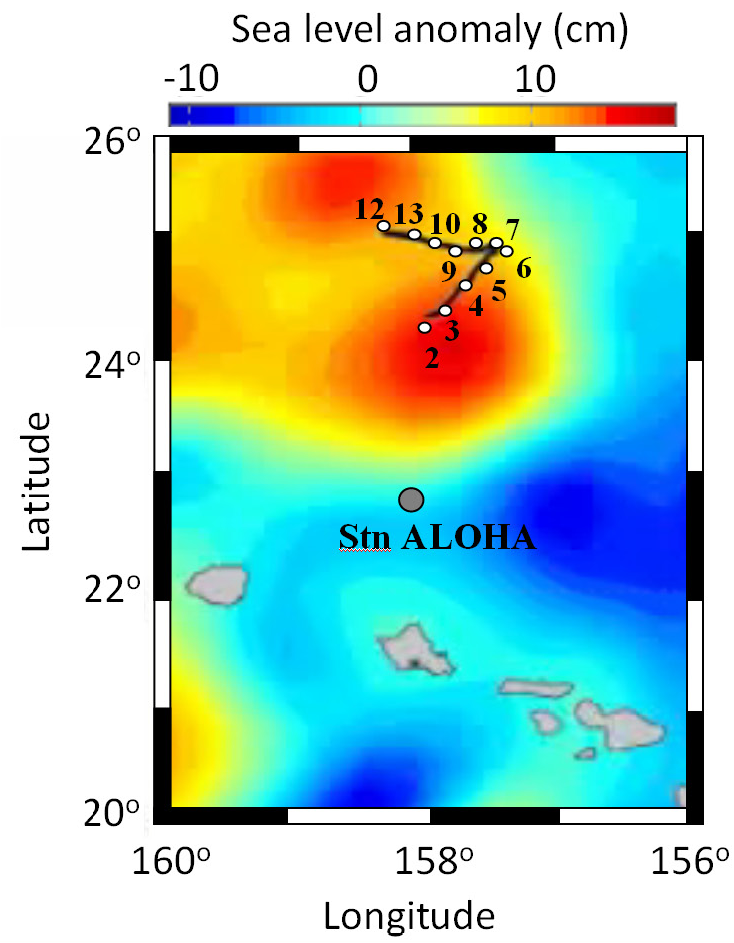

Supplement: Supplementary file 2 — Figure S1. 14-day composite of satellite derived SSHA 100 km north of the Hawaiian Islands in the Pacific Ocean between 7 and 21 September 2011 (data from Moderate Resolution Imaging Spectroradiometer). A summary of the cruise transect is indicated by the solid black line and the labeled white circles represent the sampling stations discussed in the text. Station ALOHA, the long-term sampling station for the Hawaii Ocean Time-series (HOT) programme, located at 22°45′N, 158°W is also highlighted. [file emi40005-0697-SD2.tif]

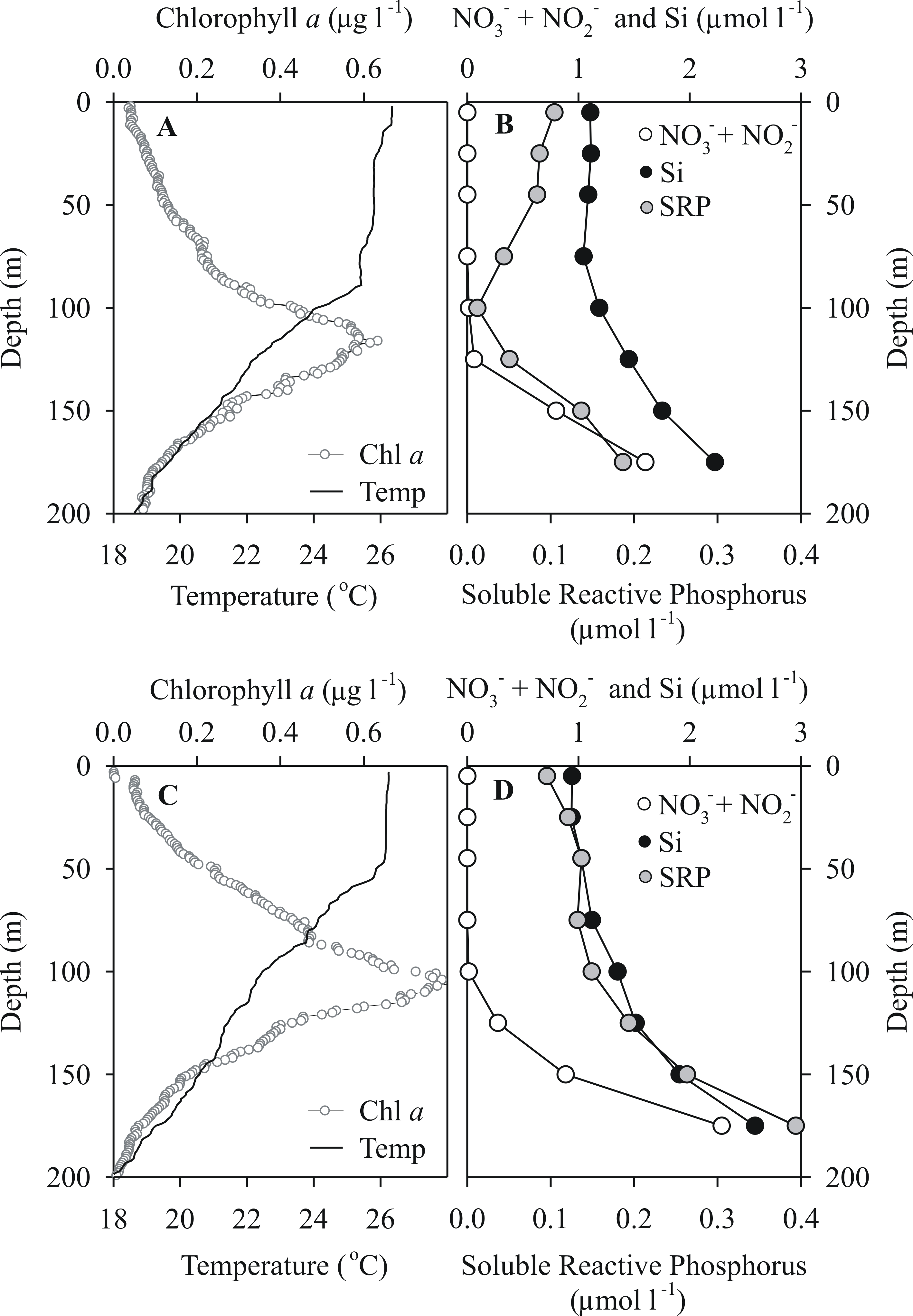

Supplement: Supplementary file 3 — Figure S2. Representative water column profiles for the two sections of the cruise track, (A-B) Stn 3 and (C-D) Stn 13. [file emi40005-0697-SD3.tif]
